# Strangulation of the umbilical cord by an amnion band - a rare cause of intrauterine demise: a case report

**DOI:** 10.1186/1757-1626-2-9108

**Published:** 2009-11-29

**Authors:** Konstantinos Chatzigeorgiou, Theodoros Theodoridis, Ioannis Efstratiou, Apostolos Athanasiadis, Leonidas Zepiridis, Filippos Tzevelekis, Basil Tarlatzis

**Affiliations:** 1 1st Department of Obstetrics and Gynecology, Aristotle University of Thessaloniki, Papageorgiou General Hospital, Ring Road, Nea Efkarpia, 56403 Thessaloniki, Greece; 2 Department of Pathology, Papageorgiou General Hospital, Ring Road, Nea Efkarpia, 56403 Thessaloniki, Greece

## Abstract

**Introduction:**

The amniotic band syndrome has a scarce prevalence and intrauterine death as a result of amniotic bands formation is extremely rare.

**Case presentation:**

We present an illustrative case of intrauterine death of an embryo in the 24th gestational week in an 30-year old primigravida. The death was ascribed to the twisting of the umbilical cord around the left upper extremity, causing a strangulation of the umbilical cord in a very impressive way.

**Conclusion:**

Constriction of the umbilical cord by an amniotic band is extremely rare and very hard, if not impossible, to diagnose with antenatal sonography.

## Background

The amniotic band syndrome, as a cause of fetal malformations, has a prevalence ranging from one in 1200 up to one in 15000 live births, and only rare cases of intrauterine fetal death by constriction of the umbilical cord have been described [1,2]. The aetiology of band formation remains unknown.

We present an illustrative case of intrauterine death of an embryo in the 24th gestational week due to constriction of the umbilical cord by an amniotic band.

## Case presentation

A 30-year-old Caucasian primigravida of Greek nationality, presented at the 18th pregnancy week. She had up to this time a fragmentary antenatal care, without first trimester sonography and without serologic evaluation for TORCH. Her medical history however, was unremarkable, without prior surgeries. During the first visit, sonographic evaluation confirmed the presence of a singleton male fetus, without obvious anatomical deformities, and fetal biometry corresponding to the 18th gestational week. She was referred for detailed fetal anatomy scan in the 23th gestational week. During this detailed scan, again no obvious deformities could be identified, but due to the position of the embryo, the heart and the upper extremities could not clearly be seen. Therefore, the patient was re-scheduled for a second visit one week later. Unfortunately, in this second visit, only intrauterine demise was ascertained, in the 23 + 4 week.

**Figure 1:**
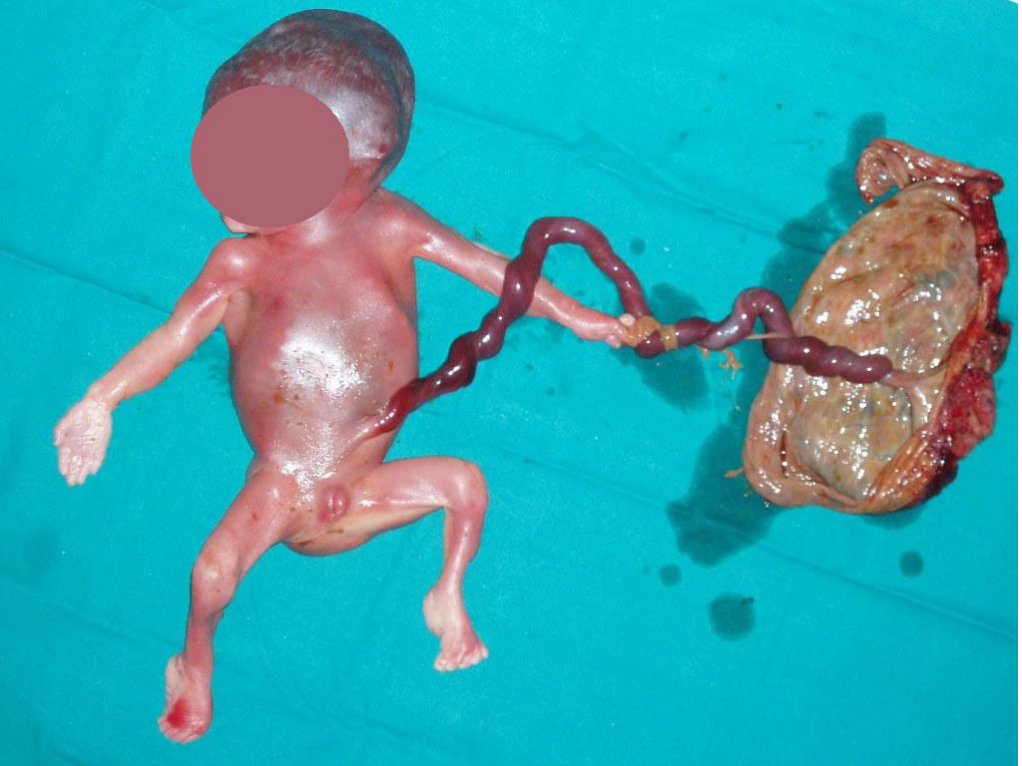
**Twisting of the umbilical cord around the left upper extremity, causing a strangulation of the umbilical cord**.

**Figure 2:**
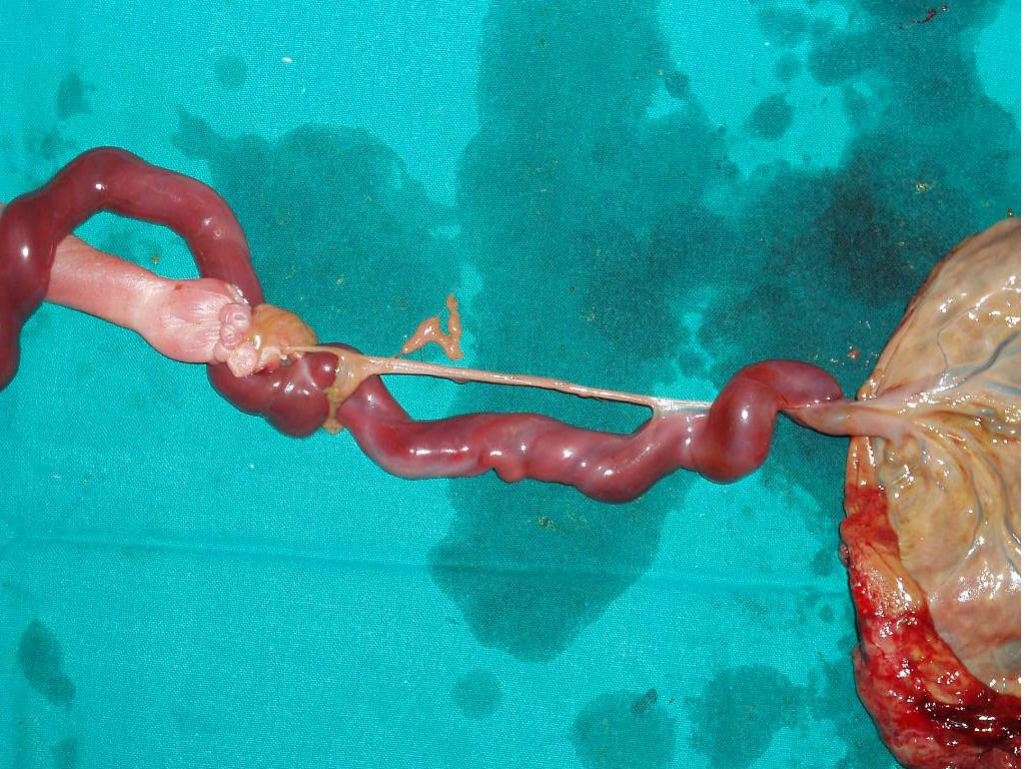
**Insertion of the umbilical cord at the hypoplastic and severely deformed left hand**.

Termination of pregnancy through induction of labour took place on the next day with vaginal 600 mcg misoprostol. A 600 g stillborn, male embryo was delivered, with a CRL of 22 cm. In approximately 12 cm from the placental insertion, the umbilical cord was twisted around the left upper extremity, causing a strangulation of the umbilical cord in a very impressive way (Figure 1). The cord inserted at the left hand which was hypoplastic and severely deformed (Figure 2). No other abnormalities could be found on autopsy. The placenta weighted 208 g and measured 14 × 9 × 2 cm, while the umbilical cord measured 30 cm and his architecture was normal with three vessels.

## Discussion

Many clinical deformities in the meaning of amniotic bands have been encountered and range from simple ring constrictions to major craniofacial and visceral defects [3]. Very often, where a band is attached to the fetus there is usually a local deformity, as described in our case [4]. However, only rare cases of intrauterine fetal death by constriction of the umbilical cord have been described in the literature, mostly in the second and third trimester [5,6] while the first description in modern literature was published in 1953, by Craven and Geddes [7].

The aetiology of the formation of amniotic bands remains controversial. The most accepted hypothesis is that of Torpin, who suggested that early amnion rupture might be the precipitating event. The amnion is torn during the first trimester and this abnormal amnion might form bands with the chorionic mesenchyma which can attach to the embryo [8].

Early diagnosis is usually not possible and might be suspected only in presence of other embryonic malformations. Elevation of β-HCG as a result of placental attempt to counteract the fetal growth restriction and hypoxia, due to the strangulation of umbilical cord by the amniotic bands, has also be described [3]. However, the practical aspect of determination of β-HCG in advanced pregnancy is questionable.

## Conclusion

Constriction of the umbilical cord by an amniotic band leading to intrauterine fetal death is extremely rare and very hard, if not impossible, to diagnose with antenatal sonography.

## Abbreviations

TORCH: Toxoplasmosis, Other Agents, Rubella, Cytomegalovirus, Herpes Simplex; β-HCG: beta - human chorionic gonadotropin.

## Consent

Written informed consent was obtained from the patient for publication of this case report and accompanying images. A copy of the written consent is available for review by the Editor-in-Chief of this journal.

## Competing interests

The authors declare that they have no competing interests.

## Authors' contributions

KC and TT participated in patient management, acquisition of data, interpretation of data, and were the major contributors in writing the manuscript. IE performed the pathology of the specimen. AA participated in patient management. LZ, FT and BT critically revised the paper. All authors read and approved the final manuscript.
